# Cardioprotection from stress conditions by weak magnetic fields in the Schumann Resonance band

**DOI:** 10.1038/s41598-018-36341-z

**Published:** 2019-02-07

**Authors:** G. Elhalel, C. Price, D. Fixler, A. Shainberg

**Affiliations:** 1 Porter School of the Environment and Earth Sciences, Faculty of Exact Sciences, Tel Aviv University, Tel Aviv, Israel; 2 Faculty of Engineering and the Institute of Nanotechnology and Advanced Materials, Bar Ilan University, Tel Aviv, Israel; 3 Faculty of Life Sciences, Bar Ilan University, Tel Aviv, Israel

## Abstract

The Schumann Resonances (ScR) are Extremely Low Frequency (ELF) electromagnetic resonances in the Earth-ionosphere cavity excited by global lightning discharges. This natural electromagnetic noise has likely existed on the Earth ever since the Earth had an atmosphere and an ionosphere, hence surrounding us throughout our evolutionary history. The purpose of this study was to examine the influence of extremely weak magnetic fields in the ScR first mode frequency range on the spontaneous contractions, calcium transients and Creatine Kinase (CK) release of rat cardiac cell cultures. We show that applying 7.8 Hz, 90 nT magnetic fields (MF) causes a gradual decrease in the spontaneous calcium transients’ amplitude, reaching 28% of the initial amplitude after 40 minutes of MF application, and accompanied with a gradual decrease in the calcium transients’ rise time. The mechanical spontaneous contractions cease after the ScR fields have been applied for more than 30 minutes, when the calcium transient’s amplitude reached ~60% of its initial value. The influence of the ScR MF was reversible, independent of the field magnitude in the range 20 pT-100 nT, and independent of the external DC magnetic field. However, the effect is frequency dependent; the described changes occurred only in the 7.6–8 Hz range. In addition, applying 7.8 Hz, 90 nT MF for 1.5 hours, reduced the amount of CK released to the buffer, during normal conditions, hypoxic conditions and oxidative stress induced by 80 μM H_2_O_2_. We show that the ScR field induced reduction in CK release is associated with a stress response process and has a protective character.

## Introduction

During our everyday life, we are surrounded by natural and manmade electromagnetic noise in a wide range of frequencies and magnitudes. The manmade electromagnetic noise is relatively new and many efforts have been made to understand its interaction with biological systems^1–3^. Natural electromagnetic noise, on the other hand, exists since the early days of Earth, thus surrounding us throughout our evolutionary history^4^. However, its influence on biological systems was poorly studied, mainly due to the low magnitude of these fields and thus lack of interest. One of the natural ELF signals is the lightning-produced Schumann Resonance (ScR). The ScR exhibit well-defined frequency peaks defined by the Earth circumference, at *f*_1_ = *7* .*8* Hz, *f*_2_ = *1*3 .*9* Hz, *f*_*3*_ = *20 *Hz, with a magnetic field intensity of a few pT^5^. The human body also produces weak alternating electromagnetic fields in the ELF range generated by excitable cells. Rat cardiomyocytes generate 1–10 Hz rhythm with a magnetic field magnitude of about 50 pT^6^. The purpose of this research was to examine the influence of the natural, frequency specific, ScR signal on rat cardiomyocyte cultures and to examine the coupling between these two natural ELF fields.

The ability of a cardiomyocyte to contract depends on the proper operation of many biological processes. Calcium ion transients are the key mediators between the mechanical contractions and the cardiac action potentials which initiate the contractions. The calcium influx and the calcium release from the Sarcoplasmic Reticulum (SR) in phase 2 of the action potential increases the free calcium concentration in the cytoplasm. This free calcium triggers the physical contraction mechanism. We therefore examined the ScR MF influence on the mechanical contractions and their triggering calcium transients as a basis for a more thorough investigation. In addition, in order to examine whether the impact is of a protective or a destructive nature, we examined the influence of ScR MF on CK release during normal, hypoxic and oxidative stress conditions. The dependency on the MF characteristics: magnitude, frequency and additional DC MF was studied in order to understand the physical mechanism behind the phenomena.

## Methods

All experiments were performed at 37 °C on 3- to 7-day-old cardiomyocyte cultures, exhibiting synchronized spontaneous contractions. All methods were carried out in accordance with relevant ethical guidelines and regulations, and experimental protocols have been approved by Bar Ilan University.

### Culture preparation

Sprague-Dawley rat hearts (1 to 2 days old) were removed under sterile conditions and washed three times in Phosphate Buffered Saline (PBS) to remove excess blood cells. The PBS composition was as follows: 135 mM NaCl, 2.7 mM KCl, 10 mM Na_2_HPO_4_, 1.8 mM KH_2_ PO_4_, 0.9 mM CaCl_2_, 0.5 mM MgCl_2_. The hearts were minced and then gently agitated in RDB (fig tree extract, Biological Institute, Ness-Ziona, Israel). RDB was diluted 1∶200 in Ca^2+^- and Mg^2+^-free PBS at 25 °C and incubated with the heart fragments for several cycles of 10 min each^7^. Dulbecco’s modified Eagle’s medium (DMEM) containing 25 mM glucose, supplemented with 10% inactivated horse serum (Biological Industries, Kibbutz Beit Haemek, Israel) and 0.5% chick embryo extract, was added to the supernatant containing the suspension of dissociated cells. The mixture was centrifuged at 300 *g* for 5 min. The supernatant was discarded and the cells were re-suspended. The cell suspension was diluted to 10^6^  cells/mL, and 1.5 mL suspension was placed in 35-mm plastic culture dishes on 25-mm microscope coverslip coated with collagen/gelatin. The cultures were incubated in a humidified atmosphere of 5% CO_2_ and 95% air at 37 °C. A confluent monolayer exhibiting spontaneous contractions developed within 2 days.

### Magnetic field application

An alternating magnetic field was applied by a single wrapping copper coil loop with a diameter of 35 mm wrapped around the culture dish while placed either on the microscope or during incubation. In the majority of the experiments, we used a sinusoidal 7.8 Hz, 2.5 mA current which according to Biot-Savart law induced a relatively uniform magnetic field in the samples area parallel to the sample plane ${B}_{z}={\mu }_{0}I/2R=90nT$. Lower magnetic field magnitudes were achieved by using 10 mVpp, 1 kΩ (180 pT) and 10 mVpp, 10 kΩ (18 pT). Sinusoidal waveforms in the frequency range 7–8.6 Hz were used in a few experiments. An additional DC power supply was connected to a copper coil wrapped around the culture dish with an 80 mm diameter to apply according to Biot-Savart law a DC magnetic field of ±10 µT in the cultures’ plate direction.

### Mechanical contraction measurements

A culture dish containing adherent cells was rinsed twice with PBS, re-suspended in 1 mLglucose-enriched PBS and attached to the stage of an inverted phase interference microscope. The video technique for contraction measurement has been described previously^8^. The movement of the cell border was monitored 400 times/sec for sections of 5 seconds. The time variation was then converted to voltage, filtered, and analyzed by the SAMPLE computer program. The rate of contractions was calculated according to the number of peaks in each measurement.

### Calcium imaging

Intracellular calcium was monitored using the Indo-1-AM dual emission indicator on a Zeiss inverted epifluorescence microscope. Cells were incubated at room temperature for 45 min in 1 mL glucose-enriched (25 mM) PBS with 3 μM Indo-1-AM (Molecular Probes, Eugene, OR, USA) and 2 μM Pluronic acid. After incubations, the cells were rinsed twice with PBS, and the coverslip was placed on the microscope chamber with 1 ml glucose-enriched PBS. The culture was excited at 340 nm and the emitted light then split by a dichroic mirror into two photomultipliers (series no. H5700/HC120, Hamamatsu, Japan), with input filters at 410 and 490 nm for Indo-1. Intracellular calcium concentration was estimated using the 410/490 ratio^9^.

### Hypoxic conditions

Cultures were washed twice from the medium with glucose-free Tyrode (137 mM NaCl, 5.4 mM KCl, 10 mM HEPES, 1.2 mM CaCl_2_, 0.5 mM MgCl_2_) at pH 7.4 and then re-suspended in 1 ml glucose-free Tyrode. The magnetic field was applied for 1.5 hours before exposing the cells to hypoxic conditions at 37 °C. The hypoxic condition consisted of 120 min in a hypoxic chamber where the atmosphere was replaced by the inert gas argon (100%).

### Oxidative stress conditions

Cultures were washed twice with glucose-enriched Tyrode at pH 7.4 and then re-suspended in 1 mL glucose- enriched Tyrode. Cells were treated with 80 μM H_2_O_2_ either during or after the magnetic field application and incubated at 37 °C in a dark environment for 60 minutes. The oxidative stress damage was characterized at the end by the release of CK to the cell medium. Two types of tests were performed: 1. samples were subjected to a magnetic field for 1.5 hours before the addition of H_2_O_2_ for an hour. 2. Samples were subjected to 0.5 hours of magnetic field and treated with H_2_O_2_ for an hour simultaneously with the magnetic field application.

### CK measurements

At the end of the experiment, 25 μL supernatant of each plate was transferred into a 96-well dish and the CK activities were determined with a CK-MB kit (Sigma), as described by the manufacturer. The product of the enzyme was measured spectrometrically at 30 °C at a wavelength of 340 nm.

## Results

### Influence of ScR MF on spontaneous mechanical contractions and calcium transients

**Figure 1:**
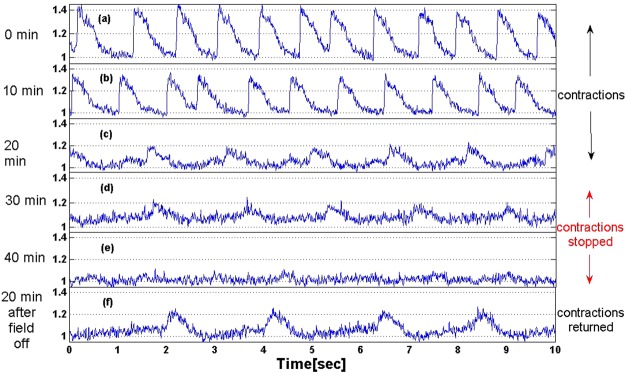
Intracellular calcium measurements of cardiomyocytes during exposure to ScR MF (7.8 Hz, 90 nT). The contractions stopped after 20 minutes whereas Ca transients vanished only after 40 min. The ScR MF stimulation was turned off after 50 minutes and intracellular calcium transients returned.

The influence of 7.8 Hz, 90 nT MF on the spontaneous calcium transients of cardiomyocytes is demonstrated in Fig. 1. Each subplot (Fig. 1a-f), displays a single 10 seconds measurement of intracellular calcium level at a different time. The spontaneous calcium transients’ amplitude, with the MF applied, decreases slowly until it almost totally disappears after 40 minutes (Fig. 1e). 25 minutes after the MF appliance, the mechanical contractions stopped, even though the calcium transients still exist with a low amplitude (Fig. 1d,e). The MF was turned off after 50 minutes of stimulation and the calcium transients’ amplitude recovered to ~50% of the initial amplitude after 20 minutes, and the contractions returned.

**Figure 2:**
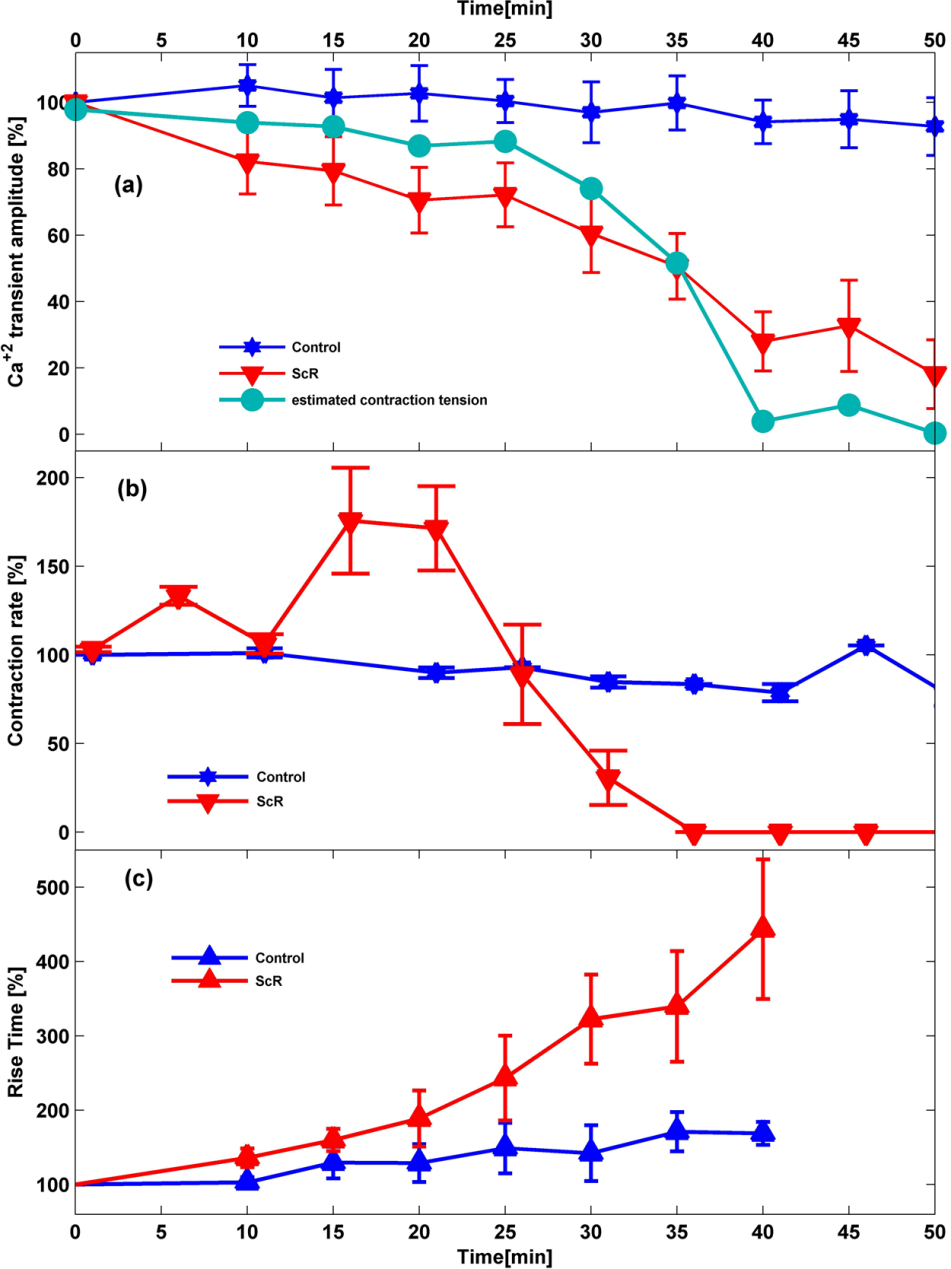
Effect of ScR MF on the amplitude of calcium transients, spontaneous contraction rate and the calcium transient rise time. (**a**) Relative spontaneous calcium transients amplitude of the control (blue stars) and ScR groups (red triangles). The turquoise curve shows the contraction tension estimated from the calcium transient amplitude. (**b**) Spontaneous mechanical contraction rate of cardiomyocytes Vs. time. Blue stars and red triangles represents the Control (no MF) and the 7.8 Hz, 90 nT MF respectively. (**c**) Relative transient rise time normalized with the transient amplitude of the control group (blue up facing triangles) and ScR group (red up facing triangles).

Figure 2a presents the relative change in the averaged calcium transient amplitude of the control (no MF, blue stars) and ScR stimulation (with the 7.8 Hz, 90 nT MF, red triangles) groups Vs. time. The relative amplitude decrease after 40 minutes of measurements was −72% in the stimulated group, compared to only −7% in the control group. Figure 2b presents the average mechanical contraction rate (spontaneous contractions) Vs. time of the control and the ScR MF groups. The mechanical contraction rate varies between samples, so all measurements were normalized to the contraction rate at the beginning of the measurement. The control group (blue curve) exhibits a relatively constant contraction rate while the ScR MF group (red curve) presents an increase in contraction rate, up to 200% of the initial contraction rate during the first 20 minutes of MF followed by a sharp decrease towards the complete suspension of the mechanical contractions after 35 minutes of MF application. The mechanical contraction tension can be estimated from the intracellular calcium transient amplitude using the Hill equation with a Hill constant of 5.6^10^. The turquoise curve in Fig. 2a presents the decrease in contraction tension estimated from the measured calcium concentration. As opposed to the gradual decrease in the transients’ amplitude, it predicts a sharp decrease from 90% of the initial tension down to only 4% in 15 minutes reaching a complete termination of the spontaneous contraction after 40 minutes with the ScR MF, similar to the measured sharp decrease and termination of the contractions after 35 minutes with the 7.8 Hz, 90 nT MF.

The influence of the ScR MF on the spontaneous calcium transients normalized rise time is presented in Fig. 2c. The rise time was normalized with the transients’ amplitude. The transient rise time of the ScR group (Fig. 2c, red up facing triangles) exhibits a 450% increase after 40 minutes with the ScR field compared to a very small increase of the control normalized rise time (blue, up facing triangles). Both, the normalized relaxation time and the diastolic calcium concentration did not display a significant difference between the ScR and control groups (data not presented).

### ScR MF induced cardioprotection in stress conditions

**Figure 3:**
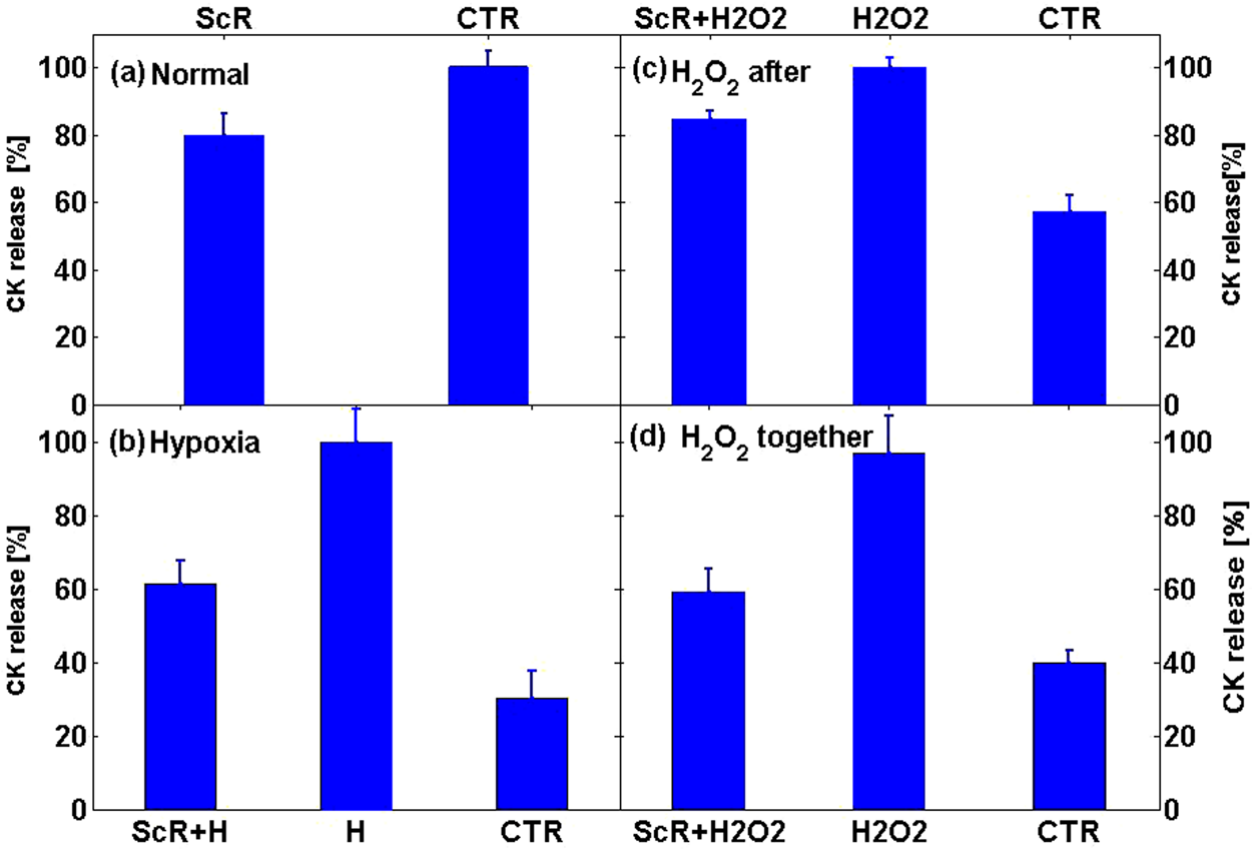
Effect of ScR MF on cardio protection as revealed by CK released from the cells. Relative CK release in (**a**) Normal condition: CTR- 1.5 hours of normal conditions, ScR- 1.5 hours of ScR MF application in normal conditions. (**b**) Hypoxic conditions: ScR + H- 1.5 hours of MF application followed by 2 hours of hypoxia, H- 1.5 of normal conditions followed by 2 hours of hypoxia. CTR- 3.5 hours of normal conditions. (**c**) H_2_O_2_ after MF: ScR + H_2_O_2_–1.5 hours of MF application followed by 1 hour with 80 µM H_2_O_2_, H_2_O_2_–1.5 hours of normal conditions followed by 1 hour with 80 µM H_2_O_2_, CTR- 2.5 hours of normal conditions. (**d**) Simultaneous application of H_2_O_2_ and ScR MF: ScR + H_2_O_2_–0.5 hours of ScR MF application followed by 1 hour with the simultaneous application of 80 µM H_2_O_2_ and ScR MF, H_2_O_2_–0.5 hours of normal conditions followed by 1 hour with 80 µM H_2_O_2_, CTR- 1.5 hours of normal conditions.

We have also examined the influence of applying the ScR MF (for 1.5 hours) on CK release in 4 different experimental configurations: (1) in normal conditions (2) When the ScR MF application was followed by 2 hours of hypoxia (3) When 80 µM of H_2_O_2_ was added to the cultures for 1 hour following the ScR MF application (4) Samples were subjected to 0.5 hours of 7.8 Hz, 90 nT MF and then 80 μM H_2_O_2_ was included to the buffer for an hour and subjected simultaneously to the MF application. Normalized CK release in all four experiments with no MF is presented in Fig. 3 (CTR group). In the hypoxic and oxidative stress experiments, CK release following the stress was 40–70% higher than in the control groups. However, in all four experimental procedures, the ScR MF application reduced the amount of CK release, suggesting a protective effect. In the normal condition experiment, spontaneous CK release was 20% lower in the ScR group (Fig. 3a). A similar reduction was seen when the ScR MF was applied before the oxidative stress (Fig. 3c). A more significant reduction (~40%) in CK release was seen in the hypoxia experiment (Fig. 3b) and when the ScR MF was applied simultaneously with the oxidative stress (Fig. 3d).

**Figure 4:**
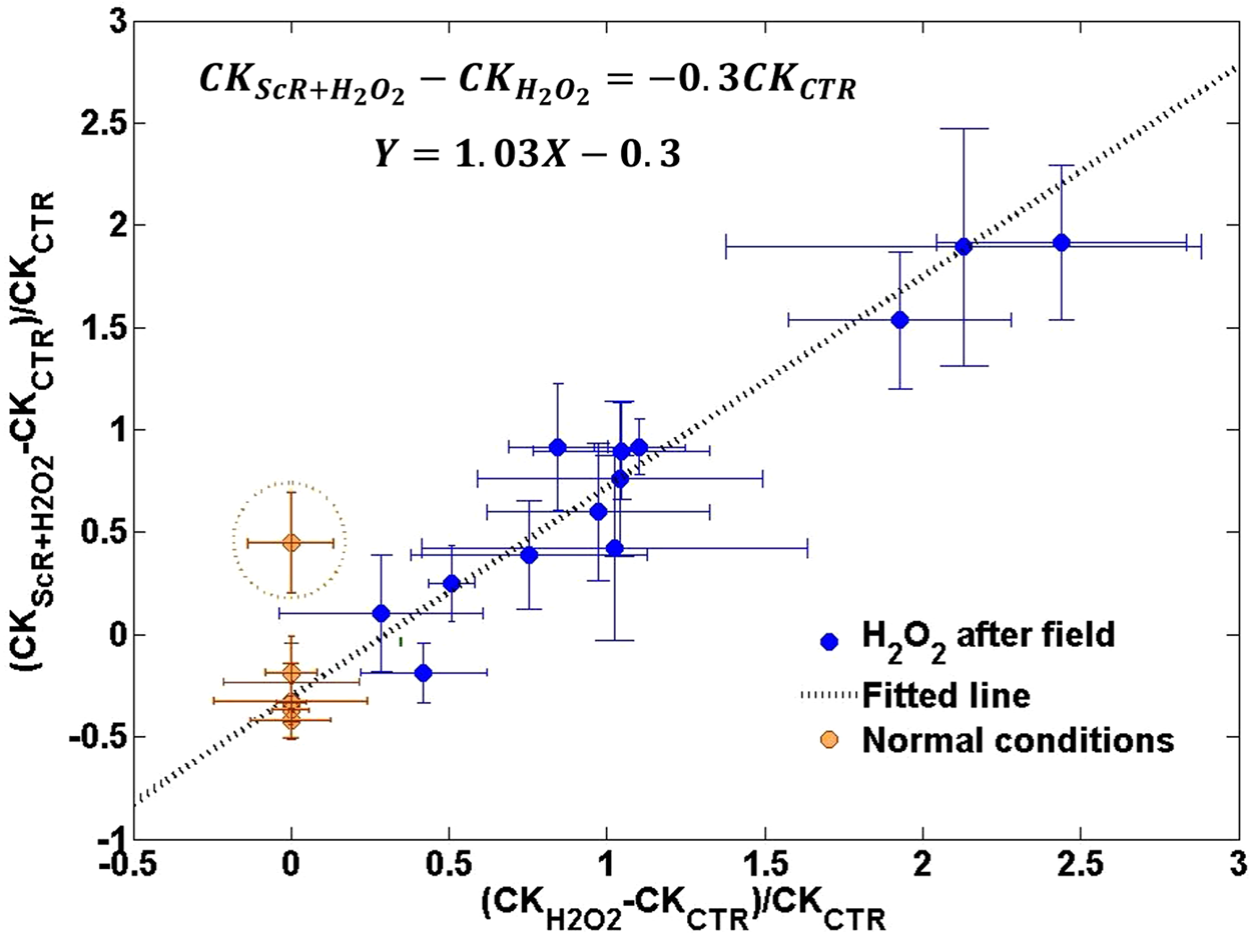
Relative “damage” following the ScR MF application Vs. the damage due to stress alone. Blue circles – oxidative stress applied following the ScR field. Yellow circles – normal conditions experiments. dashed line – linear fit.

Assuming that CK release represents the stress-induced damage, we examined the protective character of the ScR MF. We plotted the relative “damage” following the ScR MF application $(C{K}_{ScR+{H}_{2}{O}_{2}}-C{K}_{CTR})/C{K}_{CTR}$ versus the damage due to stress alone $\,(C{K}_{{H}_{2}{O}_{2}}-C{K}_{CTR})/C{K}_{CTR}$. Figure 4 presents the results of the third experiment (see Fig. 3c), when the ScR MF was applied before the oxidative stress (blue circles). Each point represents a single experiment and an average of 3–4 samples. As can be seen from the plot, the results exhibit a linear behavior with a unitary slope which translates to $C{K}_{ScR+{H}_{2}{O}_{2}}-C{K}_{{H}_{2}{O}_{2}}=-0.3C{K}_{CTR}$. It suggests that the ScR MF induced ‘protection’ depends only on the CK release in normal conditions and does not depend on the oxidative stress induced damage. To support this deduction we added the results of the normal condition experiments (yellow circles in Fig. 4) using $C{K}_{{H}_{2}{O}_{2}}-C{K}_{CTR}=0$ (no oxidative stress). Except for one experiment, all the normal conditions results fit the same unitary slope linear relation.

**Figure 5:**
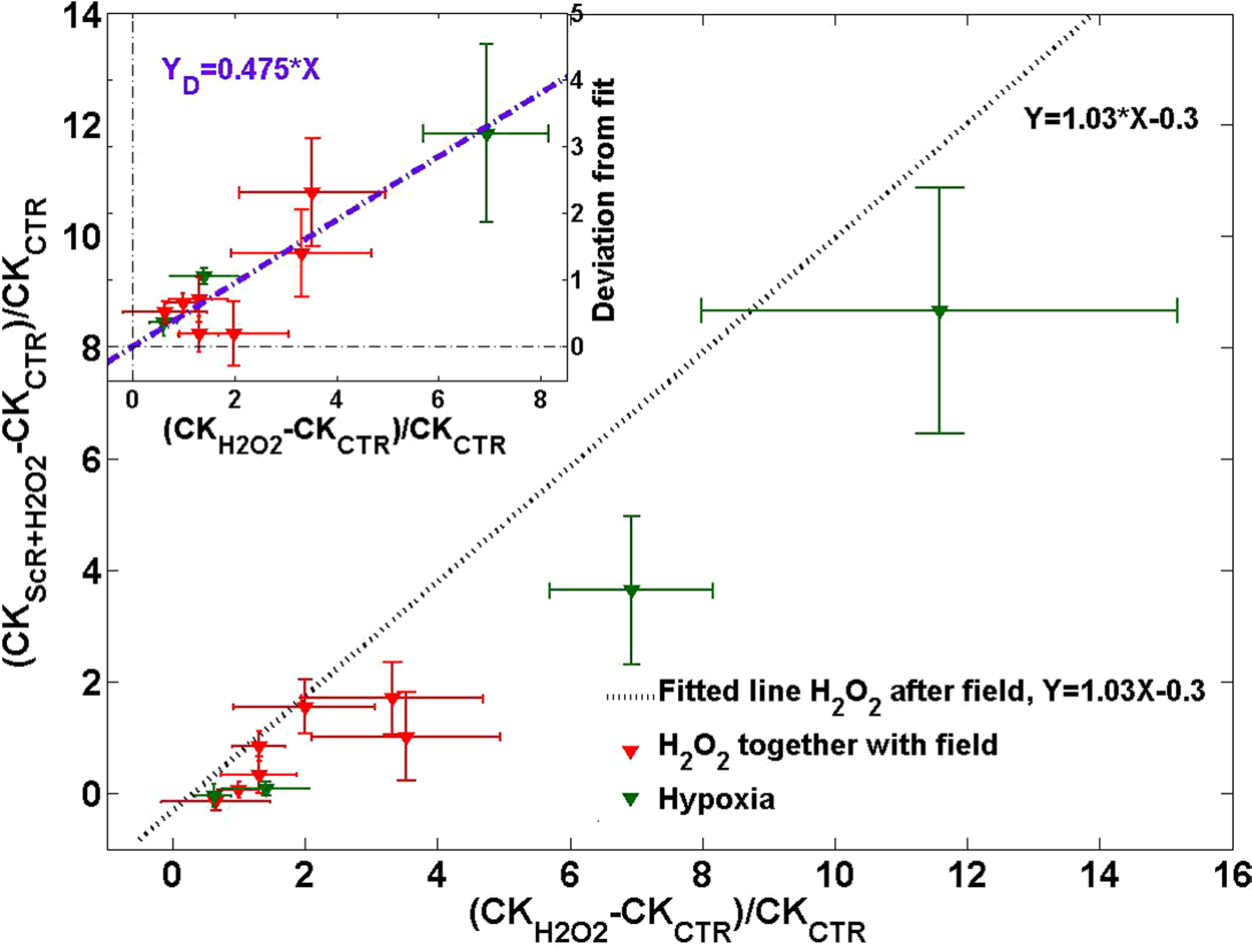
Relative “damage” following the ScR MF application Vs. the damage due to stress alone. Green triangles – Hypoxia experiment. Red triangles – oxidative stress applied simultaneously with the ScR MF. Inset – deviations from the linear normal conditions line.

The relative “damage” ratio of the fourth experiment (see Fig. 3d) and the hypoxia experiment (see Fig. 3b) is presented in Fig. 5. All the results lie below the normal conditions line $(C{K}_{ScR+{H}_{2}{O}_{2}}-C{K}_{{H}_{2}{O}_{2}}=-0.3C{K}_{CTR})$ obtained in the previous plot. The inset in Fig. 5 presents the deviations from the normal conditions line after removing the hypoxia experiment with the relatively large CK release (the influence of the ScR MF upon acute damage might be different). These deviations also fit a linear relation (from here on referred to as ‘protection line’), which passes through the origin, as expected in the no-damage-no-protection situation. The 0.475 slope of the protection line implies that almost 50% of the increase in CK release was prevented by the ScR MF.

### Dependency in the magnetic field parameters

**Figure 6:**
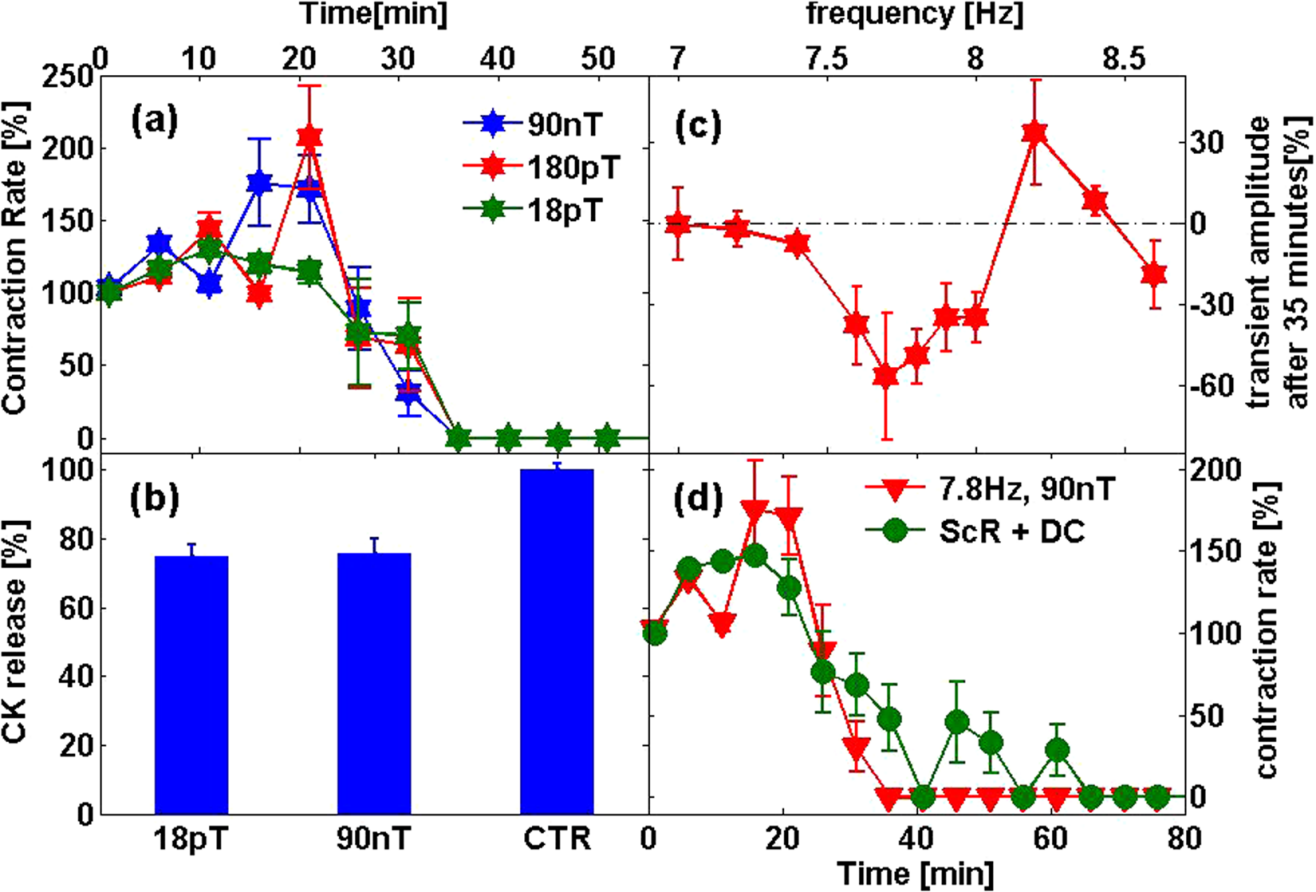
Dependency on the MF parameters. (**a**) Spontaneous contraction rate Vs. time with the magnetic field at various field magnitudes. (**b**) Comparison between the influence of 90 nT and 18 pT, 7.8 Hz magnetic field on CK release. (**c**) Relative change in calcium transients’ amplitude after 35 minutes with 90 nT magnetic field at various frequencies. (**d**) Dependency on external DC field. The influence of 7.8 Hz, 90 nT on spontaneous contraction rate with (green curve) and without (red curve) an additional ±10 μT DC field.

A few theories trying to explain the influence of weak alternating magnetic fields on biological entities were suggested^11^. Each presents a different dependency on the magnetic field characteristics: the stimulation intensity, frequency and the external magnetic DC field. In order to examine the relevance of these theories to the above phenomenon, we tested the dependence of the effect on the magnetic field parameters. Figure 6a,b presents the dependency of the mechanical contraction rate and CK release in normal conditions on the MF magnitude. Both, the reduction in mechanical contractions rate and the normal conditions CK release were similar in this magnitude range (18 pT-90 nT). Figure 6c presents the relative change in the calcium transients’ amplitude after 35 minutes with a 90 nT field at different frequencies. Only frequencies in the range 7.6–8 Hz caused a decrease of the calcium transients’ amplitude. There was no influence on the transients’ amplitude when 7–7.4 Hz and 8.4–8.6 Hz magnetic fields were applied. A large increase (~35%) was measured during the 8.2 Hz field appliance after 35 minutes with the magnetic field. The dependency of the effect on the external magnetic DC field was examined by the addition of ±10 µT DC field to the 7.8 Hz, 90 nT AC field. As can be seen in Fig. 6d, a similar decrease in the mechanical contraction rate was seen when the 10 µT DC field was applied simultaneously with the ScR MF.

## Discussion

### The link between different processes

The above findings involve three separate processes engaged in the cardiomyocyte regular function; the mechanical contraction, the calcium transients and the CK release rate. Whether a few different mechanisms or a common influence path is responsible for these effects remains an open question. The abrupt termination of the spontaneous mechanical contractions was accompanied by a gradual decrease in the calcium transients’ amplitude and a longer rise time. These two observations signify a reduction in the amount and pace of calcium entrance to the cytosol. Combined with the excellent resemblance between the time dependence of the mechanical contraction rate and the estimated contraction strength, it suggests that a reduction in the intracellular calcium is most likely the cause of the contraction suspension when the calcium transients’ amplitude decrease below a certain threshold. Another justification for the connection between these two effects is the increase in contraction rate during the first 20 minutes of field application. The increased contraction rate can be a manifestation of a reduced SR load. Increased rate due to shorter relaxation time lessens the calcium efflux and therefore counterbalances the reduction in the calcium reservoir^12–14^ and enables the cell to overcome the reduction in the calcium SR reservoir.

Both the mechanical contraction and the intracellular calcium balance strongly depends on the amount of CK in the cytoplasm, being a key player in the energy maintenance mechanism^15^. During normal conditions, CK release rate is extremely low ~5% of the content of the cell in 1.5 hours. Hence, the observed variations in CK release are unlikely to significantly influence the energy balance and impede the mechanical contractions. Another potential link is through calcium influenced exocytosis. Intracellular calcium concentration variations can influence exocytosis rate and quanta^16–18^. Hence if the ScR magnetic field influences the calcium homeostasis it can indirectly affect exocytosis and the amount of CK released to the buffer. An increased reduction in CK release rate was observed during Hypoxic and oxidative stress conditions. It suggests another possible relation due to the coupling between calcium and ROS signaling pathways^19–21^. Oxidative stress elevates the intracellular calcium concentration and increased calcium concentration activates ROS generating enzymes. Increased ROS levels were shown to cause prolonged SR leaks and Ca^2+^ depletion^22^.

### Influence on calcium homeostasis

The fact that the 7.8 Hz, 90 nT MF field did not influence the diastolic Ca^2+^ concentration and had only a minor effect on the decrease time indicates that the sizable decrease in the calcium amplitude cannot be attributed solely to a direct influence on the Sodium-Calcium Exchanger (NCX) and Sarco/Endoplasmic Reticulum Ca^2+^ ATPase (SERCA) mechanisms. The large decrease in the calcium transient amplitude and the prolonged rise time suggests an influence of the ScR MF on one of the two main mechanisms responsible for calcium entrance to the cytosol; the calcium influx (through L-type voltage-dependent calcium channels) and calcium release from the SR, triggered by the Ca^2+^ influx current. Malfunction of one of these two mechanisms should translate to a slower entrance of calcium and therefore a prolonged increase time and a lower transient amplitude. A similar reduction in the calcium transient amplitude caused by ScR MF was seen in rat skeletal muscle cultures (to be published). It suggests that calcium release from the SR is the target as “Ca induced Ca release” is not relevant in skeletal muscle.

### Influence on CK release

The termination of the mechanical contractions and the reduction of the calcium concentration during the cardiomyocyte spontaneous contraction following the appliance of the ScR field might suggest a deterioration in the cells’ proper activity. We therefore examined the influence of the ScR field on CK release to the buffer. CK release rate is slow and was immeasurable after 45 minutes (smaller than the standard deviation), in which the decrease in calcium transient amplitude occurred. The average amount of CK release is ~10% of the content of the cells in 90 minutes. Such a small change seems unlikely to significantly indicate a substantial influence on the energy balance or cell viability and impede the mechanical contractions. Additionally, in order to verify that lower CK release due to the field exposure did not result from reduced cell viability we studied the influence on cell viability using Propidium Iodide (PI) staining procedure (data not presented). PI values were consistent with CK release (i.e. lower PI values for the samples with reduced CK release) and therefore imply more viable cells due to the field application.

We showed that the reduction in CK release when the ScR MF was applied before the oxidative stress was similar to the reduction in Normal conditions, and both agree with a fit to a linear line with a unitary slope: $C{K}_{ScR+{H}_{2}{O}_{2}}-C{K}_{{H}_{2}{O}_{2}}=-\,0.3C{K}_{CTR}$. It insinuates that the reduction did not depend on the amount of damage done by the oxidative stress and that the MF effect most likely persisted throughout the oxidative stress application.

In the hypoxia experiment (Fig. 3b) and when the oxidative stress was applied simultaneously with the ScR MF (Fig. 3d) the reduction in CK release was more pronounced than when the ScR MF was applied before the oxidative stress (Fig. 3c). We showed that the field influence on CK release in Fig. 3c,d, ${\Delta }_{CK}$, can be described as the composition of two factors: ${\Delta }_{CK}={\Delta }_{Normal}+{\Delta }_{Protection}$ where ${\Delta }_{Normal}$, is the normal condition contribution described above: ${\Delta }_{Normal}=-0.3C{K}_{CTR}$, depending only on the normal conditions CK release, and the ‘protection line’ contribution: ${\Delta }_{Protection}=-0.475(C{K}_{{H}_{2}{O}_{2}}-C{K}_{CTR})$, depending only on the amount of damage caused by the hypoxia and oxidative stress. This division to two independent factors insinuates two distinct independent contributions involving two separate processes. The first may possibly be related to the interaction of the ScR MF with normal condition processes such as exocytosis, while the second, probably involves one of the cells’ protection mechanisms.

The damage dependent ${\Delta }_{Protection}$ appeared in the hypoxia experiment even though the field was applied prior to the hypoxic damage. This is as opposed to the oxidative stress results, in which the damage dependent term, ${\Delta }_{Protection}$, appeared only when the ScR MF was applied simultaneously with the oxidative stress. We attributed this discrepancy to the gradual vs. abrupt increase in ROS levels in the hypoxic and oxidative stress experiments.

### Dependency on magnetic field parameters

Due to the complexity and the multiple factors and mechanisms involved, uncovering the physical mechanism behind the ScR MF effect is a complicated task, beyond the scope of this work. However, the dependency of the effect on the applied MF parameters can point towards a magnetoreception mechanism.

Both the mechanical contractions and the CK release rate were independent of the MF magnitude. This property rules out a few of the proposed physical explanation such as stochastic resonance, the Eddy currents explanation, the ion cyclotron parametric resonance explanation in which the effects’ magnitude depends on the ratio between the oscillating and static MF amplitudes, and the radical pair recombination theory. Another option is that a certain threshold exists above which the effect is plausible. The threshold could be of a physical or biological origin.

The extremely weak amplitudes of the applied MF (18 pT-90 nT) set another constraint and eliminate some of the proposed explanations. The influence on radical pair recombination for example can produce a significant affect only at higher field magnitudes^23^ (1–10 mT, see Grissom, 1995). Stochastic resonance can amplify a signal by only a factor of 100 and is therefore less probable to be relevant to the ScR effect^24^.

Another possible explanation involves the electric fields induced by the oscillating MF. The average electric field induced in the culture by the ScR MF applied in our experiment (90 nT, 7.8 Hz MF) is $\,{E}_{90nT}=4\times {10}^{-8}V{m}^{-1}$. Elasmobranch fishes were shown to be sensitive to DC and ELF electric fields of the order of $5\times {10}^{-7}V{m}^{-1}$^25,26^, only one order of magnitude higher. Such a small magnitude difference can be leveled by simple geometric reasoning for example. Therefore, biologic interaction with the induced electric field is one of the possible explanation for the ScR magnetic field effect.

The frequency specific character of the ScR effect rejects the radical pair recombination explanation which lacks frequency selectivity mechanism. The Eddy current explanation presents a linear dependency on the fields’ frequency and thus irrelevant to the ScR effect. One of the popular theories explaining the influence of weak ELF MF s in biological tissues is the ion cyclotron effect. Others^27,28^ have examined the influence of 16 Hz 40 nT field on the spontaneous calcium transients of cardiomyocytes. They demonstrated a 75% reduction in the calcium transients’ amplitude after 30 minutes of exposer. The frequency in which the field influenced the calcium transients, varied when the DC field magnitude was varied and fitted the ion cyclotron resonance frequency of potassium ion^29^. The Earth’s geomagnetic field in Tel Aviv is around 40 μT. If the ion cyclotron frequency for a DC field of 40 μT is 7.8 Hz, shifting the DC field to 50 μT or 30 μT will shift the ion cyclotron frequency to 9.75 Hz and 5.85 Hz respectively. If the ScR field influence is due to the ion cyclotron resonance, the frequency in which the field influences the cardiomyocytes should vary respectively and the 7.8 Hz field will not influence the contraction rate. As described in the results section, this is not the case, and the 7.8 Hz magnetic field influence the spontaneous contraction rate even when an additional magnetic DC is applied, and we can therefore rule out the ion cyclotron effect.

The fact that the ion cyclotron frequency is the only characteristic resonance frequency suitable for the ScR frequency range, but irrelevant to the ScR effect, makes biological tuning such as the turtle cochlear hair cells tuning, a more probable explanation. The resonance frequency of a specific hair cell is determined by the specific number and kinetics of calcium-activated (BK) potassium channels^30,31^. This mechanism supports the electric induction hypothesis as a possible precursor. According to Bellono *et al* .^32^, the frequency sensitive electrodetection of the Elasmobranch fishes derives from a low voltage activation threshold of the voltage-gated calcium channel CaV1.3 and a decreased slope conductance of the big conductance calcium-activated potassium channel (BK). These unique characteristics give rise to membrane potential oscillations (~7 Hz) that can serve as a tuning device similar to the electrical resonance mechanism of hair cells. In cardiomyocyte, BK channels appear absent from the sarcolemma, but the channels are present in mitochondrial membranes^33^ and were shown to be involved in cardioprotection against ischemia via ROS dependent mechanism^34^. This potential explanation, involving an influence of an induced electric field on the rat mitochondria BK channels, is relevant to all of the ScR observed effects: the hypoxia, oxidative stress and calcium transients. There is some evidence showing the involvement of mitochondria BK channels in cardioprotection against ischemia via fine-tuning of the oxidative state^35–37^. Additionally, ROS production in the mitochondria was shown to regulate Ca^2+^ in rat cardiomyocytes in a bidirectional, time-dependent manner^38^. They showed that induced mitochondrial ROS production caused a transient increase in Ca^2+^ spark activity, followed by gradual spark suppression partially caused by a reduction in the SR calcium load over a time scale of 15 minutes. Hence, an influence of the ScR MF with electric induction as a coupling mechanism on the mitochondria BK channel, resulting in ROS production can result in increased contraction rate, and a gradual decrease in the calcium transient amplitude.

## Summary and Conclusions

We have studied the influence of extremely low amplitude/frequency magnetic fields at the ScR first mode frequency on fundamental cellular processes such as calcium handling and stress-induced reactions in rat cardiomyocytes cultures. We showed that extremely weak 7.8 Hz magnetic fields reduce the calcium transients amplitude and had a protective effect during oxidative stress and hypoxic conditions. The effect was independent of the magnetic field magnitude and the external magnetic DC, and was pronounced only in a narrow frequency range around the first mode of the ScR field (7.8 Hz). Demonstrating a relation between the natural ScR signal and cardiomyocytes activity requires a complete understanding of the biological influence path and the theoretical physical description, and should involve a more thorough investigation of isolated components such as membrane and SR ionic currents, and their interaction with a wider range of magnetic field parameters.

This work studied the influence of a pure sine wave with a well-defined frequency. The real Schumann Resonance signal is a superposition of many individual time-delayed signals and is therefore very different from the pure coherent experimental sine. Hence this deduction is not straightforward, and a proper investigation of the influence of the second and third ScR peaks and a more realistic signal should be performed in order to confirm the possible impact of the real ScR signal.

Another substantial difference between the real ScR signal and the magnetic field in our experiment is the magnetic field magnitude. Most experiments described above were done with magnetic field 50,000 times stronger than the actual ScR field. Nevertheless, we presented evidence that the influence of the 7.8 Hz magnetic field on contraction rate and CK release is independent of the field magnitude, and that a 7.8 Hz, 18 pT magnetic field, only one order of magnitude higher than the real SR signal, have a similar effect on the cardiomyocyte cultures. These two lines of evidence suggest that a 2 pT signal could have a similar impact on cardiomyocytes. Unfortunately, technical difficulties in isolating the experimental apparatus from the natural ScR signal prevented us from properly examining the influence of a 2 pT field in a magnetically shielded environment. These two experiments should be a part of future work as a first step to validating the influence of the real ScR signal.

## Data Availability

The datasets generated during and/or analysed during the current study are available from the corresponding author on reasonable request.

## References

[1] Tenforde TS, Kaune WT (1988). Interaction of extremely low frequency electric and magnetic fields with humans. Health physics.

[2] Tenforde TS (1991). Biological interactions of extremely-low-frequency electric and magnetic fields. Bioelectrochemistry and Bioenergetics.

[3] King, R. W. The interaction of power-line electromagnetic fields with the human body. *IEEE Engineering in Medicine and Biology Magazine*, 67–73 (1998). 10.1109/51.731324 9824765

[4] Bianchi C, Meloni A (2007). Natural and man-made terrestrial electromagnetic noise: an outlook. Annales of Geophysics.

[5] Price C, Melnikov A (2004). Diurnal, seasonal and inter-annual variations in the Schumann resonance parameters. Journal of Atmospheric and Solar-Terrestrial Physics.

[6] In-Seon K, Hyuckchan K, San A, Jong-Hyu S (2011). Measurement of Rat Magnetocardiograms by Using a High-TC SQUID Magnetometer System. *IEEE Trans* . *on Appl*. Superconductivity.

[7] Shneyvays V, Zinman T, Shainberg A (2004). Analysis of calcium responses mediated by the A3 adenosine receptor in cultured newborn rat cardiac myocytes. Cell Calcium.

[8] Zangen A, Shainberg A (1997). Thiamine Deficiency in Cardiac Cells in Culture. Biochemical Pharmacology.

[9] Grynkiewicz G, Poenie M, Tsien RY (1985). A new generation of Ca2+indicators with greatly improved fluorescence properties. The Journal Of Biological Chemistry.

[10] Yue DT, Marban E, Wier G (1994). Relationship between force and intracellular [Ca21] in tetanized mammalian heart muscle. J Gen Physiol..

[11] Binhi VN, Savin AV (2003). Effects of weak magnetic fields on biological systems. physical aspects Phys-Usp,.

[12] Bers D (2002). M. cardiac ecitation-contraction coupling. Nature.

[13] Antoons G, Mubagwa K, Nevelsteen I, Sipido KR (2002). Mechanisms underlying the frequency dependence of contraction and [Ca2+]itransients in mouse ventricular myocytes. Journal of Physiology.

[14] Trafford AW, Díaz ME, Negretti N, Eisner DA (1997). Enhanced Ca2+Current and Decreased Ca2+Efflux Restore Sarcoplasmic Reticulum Ca2+Content After Depletion. Circulation Research.

[15] De Sousa E (1999). Subcellular Creatine Kinase Alterations Implications in Heart Failure. Circulation Research.

[16] Barclay JW, Morgan A, Burgoyne RD (2005). Calcium-dependent regulation of exocytosis. Cell Calcium.

[17] Kaftan EJ, Xu T, Abercrombie RF, Hille B (2000). Mitochondria Shape Hormonally Induced Cytoplasmic Calcium Oscillations and Modulate Exocytosis. The Journal of Biological Chemistry.

[18] Pang ZP, Südhof TC (2010). Cell biology of Ca2+-triggered exocytosis. Current Opinion in Cell Biology.

[19] Görlach A, Bertram K, Hudecova S, Krizanova O (2015). Calcium and ROS: A mutual interplay. Redox Biology.

[20] Gordeeva AV, Zvyagilskaya RA, Labas YA (2003). Cross-Talk between Reactive Oxygen Species and Calcium in Living Cells. Biochemistry (Moscow).

[21] Ermak G, Davies KJ (2002). Calcium and oxidative stress: from cell signaling to cell death. Molecular Immunology.

[22] Terentyev D (2008). Redox Modification of Ryanodine Receptors Contributes to Sarcoplasmic Reticulum Ca2+Leak in Chronic Heart Failure. Circ Res ..

[23] Grissom CB (1995). Magnetic Field Effects in Biology: A Survay of Possible Mechanisms with Emphasis on Radical-Pair Recombination. Chem . Rev.

[24] Binhi VN, Savin AV (2003). Effects of weak magnetic fields on biological systems: physical aspects. Phys .-Usp.

[25] Kalmijn AJ (2000). Detection and Processing of Electromagnetic and Near-Field Acoustic Signals in Elasmobranch Fishes. Philosophical Transactions: Biological Sciences.

[26] Kramer, B. Electroreception and Communication in Fishes. *Progress in Zoology*, 42. (Gustav Fischer, 1996).

[27] Yitzhaki S, Shainberg A, Shaked M, Schuss Z, Fixler D (2011). Weak Magnetic Field at 16 Hz Affects Cardiac Myocyte Ca2+transients and Reduces Cells Damage caused by Hypoxia. The Open Optics Journal.

[28] Fixler D, Yitzhaki S, Axelrod A, Zinman T, Shainberg A (2012). Correlation of Magnetic AC Field on Cardiac Myocyte Ca2 Transients at Different Magnetic DC Levels. Bioelectromagnetics.

[29] Adler D, Fixler D, Scheinowitz M, Shainberg A, Katz A (2016). Weak electromagnetic fields alter Ca2+handling and protect against hypoxia-mediated damage in primary rat skeletal muscle cultures. Pflugers Arch - Eur J Physiol.

[30] Fettiplace R, Fuchs PA (1999). Mechanisms of hair cell tuning. Annu . Rev . Physiol ..

[31] Fettiplace R (2017). Hair Cell Transduction, Tuning, and Synaptic Transmission in the Mammalian Cochlea. Compr . Physiol ..

[32] Bellono NW, Leitch DB, Julius D (2017). Molecular basis of ancestral vertebrate electroreception. Nature.

[33] Bentzen BH, Olesen S-P, Rønn LB, Grunnet M (2014). BK channel activators and their therapeutic perspectives. Frontiers in physiology.

[34] Borchert GH, Hlavackova M, Kolar F (2013). Pharmacological activation of mitochondrial BKCa channels protects isolated cardiomyocytes against simulated reperfusion-induced injury. Experimental Biology and Medicine.

[35] Cordeiro B, Terentyev D, Clements RT (2015). BKCa channel activation increases cardiac contractile recovery following hypothermic ischemia/reperfusion. Am J Physiol Heart Circ Physiol.

[36] Sakamoto K (2008). O. S. A novel opener of large-conductance Ca2+-activated K+(BK) channel reduces ischemic injury in rat cardiac myocytes by activating mitochondrial K(Ca) channel. J Pharmacol Sci ..

[37] Soltysinska, E. *et al*. KCNMA1 encoded cardiac BK channels afford protection against ischemia-reperfusion injury. *PLoS One*, **9**(7) (2014). 10.1371/journal.pone.0103402 PMC4114839 25072914

[38] Yan Y (2007). Bidirectional regulation of Ca2+sparks by mitochondria-derived reactive oxygen species in cardiac myocytes. Cardiovascular Research.

